# Circulating MicroRNA-486 and MicroRNA-146a serve as potential biomarkers of sarcopenia in the older adults

**DOI:** 10.1186/s12877-021-02040-0

**Published:** 2021-01-30

**Authors:** Huang-Chun Liu, Der-Sheng Han, Chih-Chin Hsu, Jong-Shyan Wang

**Affiliations:** 1 Department of Physical Medicine and Rehabilitation, National Taiwan University Hospital, Bei-Hu Branch, Taipei, Taiwan; 2 Graduate Institute of Rehabilitation Science, College of Medicine, Chang Gung University, Tao-Yuan, Taiwan; 3 Department of Physical Medicine and Rehabilitation, Keelung Chang Gung Memorial Hospital, Keelung, Taiwan; 4 Research Center for Chinese Herbal Medicine, College of Human Ecology, Chang Gung University of Science and Technology, Tao-Yuan, Taiwan; 5 Heart Failure Research Center, Keelung Chang Gung Memorial Hospital, Keelung, Taiwan

**Keywords:** Aging, microRNA, Sarcopenia, Cytokine

## Abstract

**Background:**

Age-related sarcopenia meaningfully increases the risks of functional limitations and mortality in the older adults. Although circulating microRNAs (c-miRNAs) are associated with aging-related cellular senescence and inflammation, the relationships between c-miRNAs and sarcopenia in the older adults remain unclear. This study investigates whether circulating myo-miRNAs and inflammation-related miRNAs are associated with sarcopenia in the older adults.

**Methods:**

This investigation recruited 77 eligible subjects (41 males and 36 females) from 597 community-dwelling older adults, and then divided them into normal (*n* = 24), dynapenic (loss of muscular function without mass, *n* = 35), and sarcopenic groups (loss of muscular function with mass, *n* = 18). Moreover, myo- (c-miRNA-133a and c-miRNA-486) and inflammation- (c-miRNA-21 and c-miRNA-146a) related miRNAs, as well as, inflammatory-related cytokine and peroxide levels in plasma were determined using quantitative polymerase chain reaction and ELISA, respectively.

**Results:**

Sarcopenic group exhibited lesser skeletal muscle mass index (SMI), handgrip strength, and gait speed, as well as, lower c-miR-486 and c-miR-146a levels, compared to those of normal and dynapenic groups. Moreover, c-miR-486 level was positively related to SMI (r = 0.334, *P* = 0.003), whereas c-miR-146a level was positively associated with SMI (*r* = 0.240, *P* = 0.035) and handgrip strength (*r* = 0.253, *P* = 0.027). In the receiver operating characteristic analysis for predicting sarcopenia, the area under the curve in c-miR-486 was 0.708 (95% confidence interval: 0.561–0.855, *P* = 0.008) and c-miR-146a was 0.676 (95% CI: 0.551–0.801, *P* = 0.024). However, no significant relationships were observed between SMI/handgrip strength/gait speed and plasma myeloperoxidase/interleukin-1훽/interleukin-6 levels.

**Conclusions:**

Myo-miRNA (c-miR-486) and inflammation-related miRNA (c-miR-146a) are superior to inflammatory peroxide/cytokines in plasma for serving as critical biomarkers of age-related sarcopenia.

## Background

Sarcopenia is defined as a loss of muscle mass and either a loss of muscle strength or physical performance [7]. Age-related sarcopenia meaningfully decreases the quality of life, leading to a loss of independence, ultimately increasing morbidity and mortality in the older adults [7, 18]. Handgrip strength has been reported to be a useful predictor of whole-body muscular strength, further applications to predict health conditions in older adults [7, 13, 18, 21]. Multi-factorial mechanisms contribute to age-declined handgrip strength, including muscular senescence, sedentary lifestyle, poor nutritional status, hormonal dysregulation, and pro-inflammation status ([11, 26, 27].

MicroRNAs (miRs) play important roles in age-related changes in muscle mass and strength, including cellular proliferation, differentiation, metabolism, and inflammation responses [16, 33]. Recent investigations have demonstrated that muscle-related microRNAs (myo-miRs) and inflammation-related miRs might be useful for estimating physical performance [16] and health conditions [11, 17].

. Moreover, circulating microRNAs (c-miRs) have been discovered in the bloodstream and bodily fluids, mature miRs can be packaged in micro-particles (exosomes, micro-vesicles, and apoptotic bodies) or complexed with miRNA-binding proteins including Argonaute 2 or high-density lipoproteins [5, 16]. Therefore, c-miRs have important functions as intercellular communication and the potential to function as the biomarkers of a physiopathological state [5, 28]. Although c-miRs are associated with aging-related processes such as cellular inflammation and senescence [2, 20], the relationship between c-miRs and muscle function in the aging process remains unclear. Accordingly, we hypothesized that myo- or inflammation-related c-miRs are associated with muscle mass and strength or physical performance in the older adults.

To answer the abovementioned questions, we compared the differences of circulating microRNAs (c-miRNAs) between dynapenia and sarcopenia in the older adults, which reflect the age-related loss of muscle quantity and quality. The loss of muscular strength or physical performance without mass serves as an early-stage of sarcopenia, while the loss of muscular strength and physical performance with mass likes to late-stage of sarcopenia. Hence, this study evaluated sarcopenia-related parameters (body composition, handgrip strengths, and gait speed) in the older adults. Furthermore, myo-miRs (c-miR-133a and c-miR-486) and inflammation-related miRs (c-miR-21 and c-miR-146a), as well as, plasma inflammatory-related peroxide and cytokine levels were determined, respectively. The present study aims to establish critical biomarkers for dynapenia and sarcopenia in the older adults.

## Methods

### Participants

**Fig. 1:**
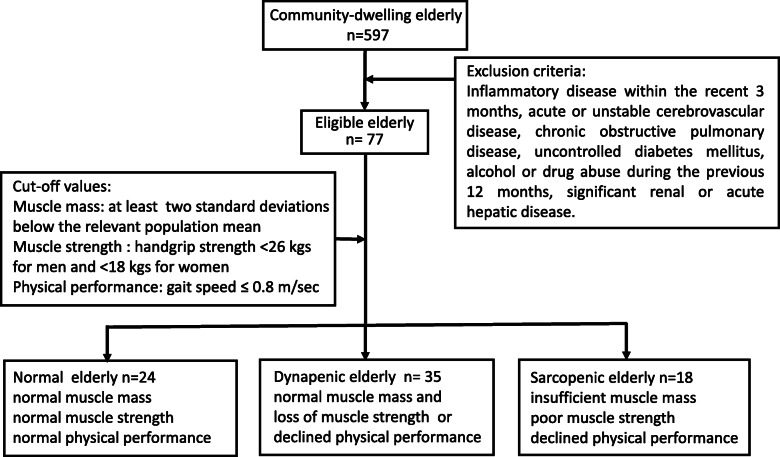
Flowchart of enrolled community-dwelling older adults included normal, dynapenic, and sarcopenic subjects during following-up. This study surveyed 597 participants who were recruited community-dwelling older adults. Exclusion criteria listed in the figure were used to recruit eligible candidates. Afterwards, eligible 77 subjects were enrolled into this study, and then divided into three groups: normal (*n* = 24), dynapenic (*n* = 35), and sarcopenic groups (*n* = 18)

This study surveyed 597 participants who were recruited community-dwelling older adults from March 2016 to December 2019 at National Taiwan University Hospital, Bei-Hu Branch, Taipei, Taiwan. Exclusion criteria included the presence of inflammatory disease within the recent 3 months, acute or unstable cerebrovascular disease, chronic obstructive pulmonary disease, uncontrolled diabetes mellitus, alcohol or drug abuse during the previous 12 months, significant renal or acute hepatic disease. The physician asks the subject’s past medical history and the subject fills out the questionnaire to exclude inappropriate subjects. Afterward, eligible 77 subjects were enrolled in this study and then divided into three groups: normal (N, *n* = 24), dynapenic (D, *n* = 35), and sarcopenic groups (S, *n* = 18) (Fig. 1). Dynapenia and sarcopenia were classified using the Asian Working Group of Sarcopenia (AWGS) criteria: (i) dynapenia is normal muscle mass and the loss of muscle strength (handgrip strength) or declined physical performance (gait speed) and (ii) sarcopenia is a condition characterized by insufficient muscle mass, poor muscle strength (handgrip strength), or/and declined physical performance (gait speed) [15]. Additionally, the N group was carefully selected to recruit the older adult subjects who were normal values in handgrip strength, gait speed, and muscular mass. The study was conducted according to the guidelines of the Declaration of Helsinki. The study was approved by the ethics committees of the National Taiwan University Hospital and all subjects provided written informed consent before participation.

### Sarcopenic parameters

#### Grip strength

Subjects’ handgrip strengths were evaluated using the analogue isometric dynamometer (BASELINE® Hydraulic Hand Dynamometer; Fabrication Enterprises Inc., White Plains, NY, USA). Appropriate position as recommended by the American Society of Hand Therapists protocols was used [4]. Measure the maximum grip strength three times, and take the best one as the result was calculated. According to the recommendation of AWGS operational definitions, cut-off values for handgrip strength (< 26 kgs for men and < 18 kgs for women) to clinical define poor muscle strength [15].

#### Gait speed

Gait speed time was assessed with a 5-m walk test, walking time was measured for all subjects over a 5-m distance as quickly as possible. Subjects were allowed to use their own walk aids during the test. Total measured three times, used the fastest time to calculate gait speed in distance (meters) divided by walking time (sec) [30]. According to the recommendation of AWGS, a low physical performance cut point is defined as a gait speed ≤0.8 m/sec.

#### Body composition

The muscle mass and body composition were determined using dual-energy x-ray absorptiometry (DXA; Stratos dR; DMS Inc., Maugio, France). Scan acquisition and analysis were performed according to manufacturer guidelines. The measurements included total body fat and lean mass was reported. Moreover, percentages of body lean (PBL) and fat (PBF), lean mass index (LMI, total lean mass/ heigh t[3]), skeletal muscle mass index (SMI, appendicular muscle mass/ heigh t[3]), and bone mass density (T score) were determined using dual-energy X-ray absorptiometry, respectively.

### Plasma sampling and RNA extraction

To minimize any possible diurnal effects., we ask all subjects to arrive at the testing center at 09:00 am. Participants were asked to fast for at least 8 h and avoid strenuous physical exercise for at least 48 h before blood sampling. Ten mL of venous blood samples were drawn from forearm venipuncture into a polypropylene tube that contained 4 mM ethylenediaminetetraacetic acid (EDTA, Sigma). Centrifugation at 1500 g for 20 min at 4 °C to obtain the plasma sample. The plasma samples were then stored in 500 μl aliquots at-80 °C, until RNA extraction. Afterward, a constant input amount of 200 μl plasma was used for RNA extraction. Synthetic *C. elegans* cel-miR-39-3p (Concentration 0.5 fM, determined by dilution series) use for spike-in control. Before the extraction of RNA, cel-miR-39 was added to all samples to monitor the efficiency and quality of the RNA extraction and qPCR procedure. The total RNA was extracted from plasma samples using a Direct-zol™ RNA MiniPrep Kit (Zymo Research, Irvine, CA, USA) according to the manufacturer’s protocol.

### Reverse transcription and c-miRNA quantification

This study evaluated four c-miRs into two categories: myo-miRs (c-miR-486-5p and c-miR-133a-3p) and inflammation-related miRs (c-miR-146a-5p and c-miR-21-5p). RNA template was used a fixed amount (1 μL) in Reverse Transcription (RT). Sample miRNAs were transcribed into cDNA via miRNA specific reverse transcription reaction using miRNA specific stem loop-RT primer and SuperScript™ III Reverse Transcriptase Kit (Invitrogen, Carlsbad, CA, USA). To quantify levels of c-miRNA, real-time quantitative polymerase chain reaction (RT-qPCR) using short Locked Nucleic Acid (LNA) probe for microRNAs specific forward primer, besides, using a universal reverse primer be the microRNAs reverse primer. Amplifications were conducted following the manufacturer’s instructions using a LightCycler® 96 Real-Time PCR System (Roche, Mannheim, Germany) [9]. For analysis quality, the acceptable Ct numbers are less than 35 and each qPCR reaction was performed in triplicate. To the estimated ratio of circulating hsa-miRs repeat cycle number to a cel-miR- 39-3p cycle number (spike-in control), formula (Ct (c-miRs assay) /Ct (cel-miR- 39 assay)) were use. In this formula, the ratio of the c-miRs is the relative expression. In addition, quantification of c-miRNAs in plasma samples were evaluated by measuring endogenous myo-miRs (c-miR-486-5p and c-miR-133a-3p) and inflammation-related miRs (c-miR-146a-5p and c-miR-21-5p) levels, as well as, exogenous control cel-miR-39 levels that was spiked in all the samples at the same concentration, method as described by Sourvinou and colleagues [25].

### Inflammation-related cytokines and lipid peroxide in plasma

Obtain 5-mL blood samples from all subjects for inflammation-related cytokines and lipid peroxide assay and placed in a centrifuge EDTA tube (final concentration, 4 mM), and immediately centrifuged at 1500 g for 10 min at 4 °C. The plasma samples were then stored in 500 μl aliquots at − 80 °C until ELISA assay. Plasma myeloperoxidase (MPO) (Immunology Consultants Laboratory, Newberg, OR), interleukin-1β (IL-1β) (eBioscience, San Diego, CA), and interleukin-6 (IL-6) (eBioscience, San Diego, CA) concentrations were quantified by commercially available ELISA kits.

### Statistical analysis

Subjects characteristics are presented in the text as means± standard deviation (SD). Data were normally distributed; data was assessed using Shapiro–Wilk tests for dependent variables. The differences in plasma c-miRs, cytokine, and sarcopenic parameters among the normal, dynapenic, and sarcopenic groups were compared by one-way ANOVA followed by Bonferroni’s post hoc test. Pearson’s correlation coefficient was used to measure the strength of the association between variables. Linear regression analysis was conducted to determine the relationship of c-miRs to handgrip strength, gait speed, and body composition. The receiver operating characteristic (ROC) curve analysis was constructed using the expression values with c-miRs and sarcopenia to distinguish between normal and sarcopenic subjects. The area under the curve (AUC) was estimated to assess the diagnostic performance of c-miRs and sarcopenia. The α level for statistical significance was set at *p* < 0.05. Data were analyzed using IBM SPSS Statistics for Windows Version 21 (IBM Corp., Armonk, NY).

## Results

### Participant characteristics

This study enrolled eligible 77 participants (66–92 (78.7 ± 6.2) years old, 41 males and 36 females) from 597 older adults (Fig. 1). Both D and S group had higher PBF (*P* < 0.05) while only the S group exhibited lower PBL (*P* < 0.05), compared to those of the N group. Moreover, the S group exhibited a smaller BMI (*P* < 0.01), LMI (*P* < 0.001) and T score (*P* < 0.01) than those of the N groups. On the other hand, the BMI (*P*<0.001), waistline (*P* < 0.001) and LMI (*P*<0.001) in the S group were inferior than those in the D group (Table 1). The effects of sarcopenic parameters and c-miRNAs, in total participants the S group exhibited a smaller SMI (*P* < 0.001), grip strength (*P* < 0.001), gait speed (*P* < 0.001), c-miR-486 (*P* < 0.05), and c-miR-146a (*P* < 0.05) than those of the N groups. In addition, the S group exhibited a smaller SMI (*P* < 0.001), grip strength (*P* < 0.001), gait speed (*P* < 0.001), and c-miR-486 (*P* < 0.05) than those of the D groups. Moreover, only the Grip strength (*P* < 0.001) a smaller exhibited in the D group than in the N group. The characteristics of males and females have similar results in sarcopenic parameters and c-miRNAs, indicating that sex is not the main influence factor in sarcopenia (Table 2).

**Table 1:** Demographic and clinical characteristics in various groups

Total *n*=77		N	D	S
Age	y/o	75.8±6.1	80.2±5.7	79.8±5.9
Sex	♂:♀	16:8	17:18	8:10
BMI	kg/m^2^	23.9±2.2	25.3±2.6	21.6±2.6^#+^
Waistline	cm	84.2±7.8	86.1±6.9	77.9±8.1^#+^
SBP	mmHg	127±14	140±14*	135±15^#^
DBP	mmHg	76±10	76±10	75±16
PBL	%	65.7±4.3	63.2±4.7	62.6±4.8^#^
PBF	%	30.1±4.7	32.9±5.2*	33.4±5.1^#^
LMI	kg/m^2^	15.7±1.7	15.5±1.7	13.5±1.4^#+^
T-score	unit	0.25±0.91	-0.39±1.03*	-0.53±0.92^#+^

*N* normal group (*n*=24), *D* dynapenic group (*n*=35), *S* sarcopenia group (*n*=18); *BMI* body mass index, *SBP* systolic blood pressure, *DBP* diastolic blood pressure, *PBL* percentage of body lean, *PBF* percentage of body fat, *LMI* lean mass index. Values are mean ± standard deviation. *P < 0.05, N vs. D; ^*#*^ *P* < 0.05, N vs. S. ^+^*P* < 0.05, D vs. S

**Table 2:** Effects of sex in sarcopenic parameters and c-miRNAs

Total *n*=77		N	D	S
SMI	kg/m^2^	7.1±0.7	7.0±0.8	5.8±0.6+#
Grip strength	kg	29.0±7.5	17.5±5.2*	10.6±5.2+#
Gait speed	m/sec	1.09±0.21	1.04±0.27	0.64±0.12+#
c-miR-486	ratio	1.09±0.04	1.08±0.04	1.06±0.04+#
c-miR-133a	ratio	1.28 ±0.08	1.28±0.07	1.26±0.05
c-miR-146a	ratio	1.24±0.06	1.23±0.06	1.20 ±0.05#
c-miR-21	ratio	1.19±0.05	1.17±0.05	1.16±0.04
Female *n*=36				
SMI	kg/m^2^	6.9±0.7	7.0±0.6	5.6±0.61+#
Grip strength	kg	28.3±8.6	17.8±5.6*	10.0±4.5+#
Gait speed	m/sec	1.22±0.24	1.10±0.24	0.61±0.13+#
c-miR-486	ratio	1.08±0.03	1.08±0.04	1.08±0.05
c-miR-133a	ratio	1.28 ±0.05	1.28±0.06	1.28±0.1
c-miR-146a	ratio	1.22±0.06	1.22±0.06	1.22 ±0.05
c-miR-21	ratio	1.18±0.05	1.17±0.05	1.18±0.05
Male *n*=41				
SMI	kg/m^2^	7.2±0.8	7.0±0.9	6.0±0.6+#
Grip strength	kg	30.1±6.9	17.2±4.9*	11.1±5.9+#
Gait speed	m/sec	0.91±0.13	1.06±0.12	0.67±0.11+#
c-miR-486	ratio	1.10±0.05	1.08±0.04	1.04±0.05
c-miR-133a	ratio	1.28±0.06	1.28±0.11	1.24±0.05
c-miR-146a	ratio	1.26±0.08	1.23±0.03	1.18±0.07
c-miR-21	ratio	1.20±0.06	1.18±.003	1.14±0.07

*N* normal group (*n*=24), *DS* decrease of strength group (*n*=35), *S* sarcopenia group (*n*=18), *SMI* skeletal muscle mass index, Values are mean ± standard deviation. *P < 0.05, N vs. D; +*P* < 0.05, D vs. S; *# P* < 0.05, N vs. S

### Sarcopenic parameters and C-miRs

**Fig. 2:**
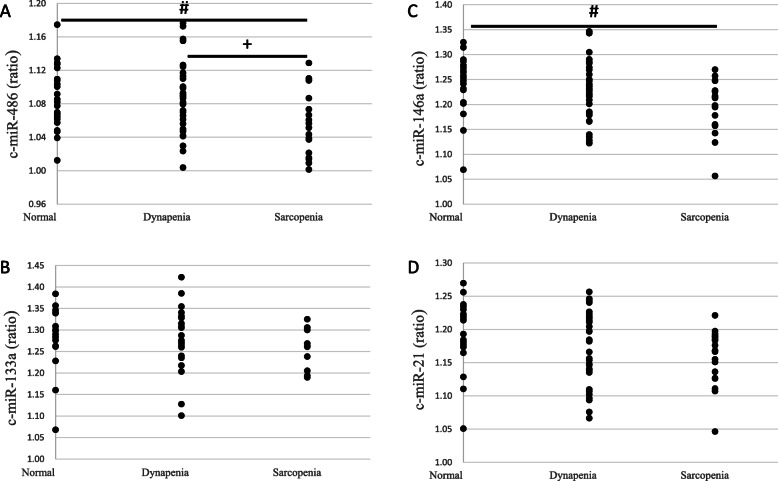
Comparisons of circulating microRNAs ((A) c-miR-486, (B) c-miR-133a (*n* = 53, due to Ct number > 35), (C) cmiR-146a, and (D) c-miR 21) among various groups. N, normal group (*n* = 24); D, dynapenic group (*n* = 35); S, sarcopenia group (*n* = 18). *P < 0.05, N vs. D; # *P* < 0.05, N vs. S. +*P* < 0.05, D vs. S

**Fig. 3:**
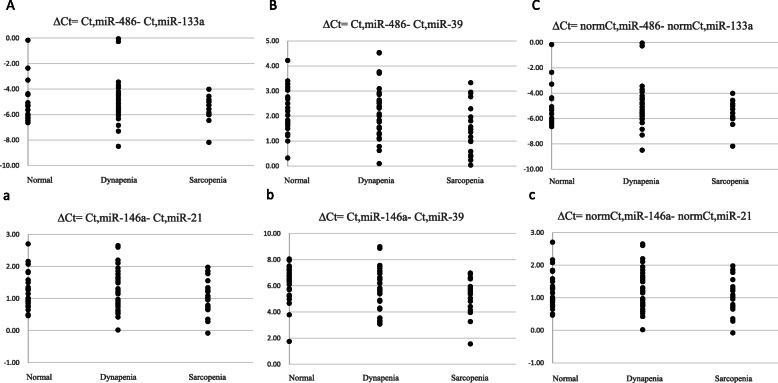
Different normalization procedures of circulating miRNA levels are no significant changes. N, normal group (*n* = 24); D, dynapenia group (*n* = 35); S, sarcopenia group (*n* = 18). The concentration of c-miR-486 and c-miR-146a in plasma was quantified by normalizing with respect to c-miR-133a (*n* = 53, due to Ct number > 35) and c-miR-21(A;a), exogenous control cel-miR-39 (B;b), and a combination of both (C;c). *P* < 0.05

Compared to the N group, both D and S groups had lesser grip strength (*P*<0.01) while the S group alone exhibited slower gait speed (*P*<0.01) (Table 2). Moreover, the S group had lower c-miR-486 (*P* < 0.05) (Fig. 2a) and c-miR-146a (*P* < 0.05) (Fig. 2c), as well as, the D group had lesser c-miR-146a (*P* < 0.05) (Fig. 2c) than the N group did. Additionally, the SMI (*P*<0.01), grip strength (*P* < 0.01), gait speed (*P*<0.01) (Table 2), and c-miR-486 *(P* = 0.023) (Fig. 2a) in the S group were inferior than those in the D group. In addition, the c-miR-486 and c-miR-146a normalizing with respect to c-miR-133a and c-miR-21, used as an endogenous control (Fig. 3a); exogenous control cel-miR-39(Fig. 3b); and a combination of the two, and then ΔCt was estimated but were no significant changes (Fig. 3c).

### Plasma lipid peroxide and inflammatory cytokines

There were no significant differences in plasma MPO, IL-1β, and IL-6 levels among the N, D, and S groups (Table 3).

**Table 3:** Plasma lipid peroxide and inflammatory cytokines in various groups

Total *n*=77		N	D	S
MPO	ng/mL	6.68±3.04	7.04±3.28	7.58±5.47
IL-1β	pg/mL	0.46±0.44	0.74±0.29	0.81±0.45
IL-6	pg/mL	6.05±2.00	5.24±1.51	5.94±2.10

*N* normal group (*n*=24), *D* dynapenic group (*n*=35), *S* sarcopenia group (*n*=18), *MPO* myeloperoxidase, *IL-1β* interleukin-1β, *IL-6* interleukin-6. Values are mean ± standard deviation

### Associations between C-miRs and Sarcopenic parameters

Pearson’s correlation coefficient was used to analyze the association between the c-miRNAs and sarcopenic variables. This present study observed that BMI, LMI and SMI were positively associated with c-miR-486 level (*P* < 0.05, Table 4). Moreover, SMI and handgrip strength were directly related to c-miR-146a level (*P* < 0.05, Table 4).

**Table 4:** Correlations of microRNAs and sarcopenic parameters

	c-miR-133a	c-miR-486	c-miR-21	c-miR-146a	BMI	Waistline	PBL	PBF	LMI	SMI	T-score	Grip strength	Gait speed
c-miR-133a	—												
c-miR-486	.420*	—											
c-miR-21	.576*	.441*	—										
c-miR-146a	.578*	.361*	.934*	—									
BMI	-.041	.253*	.023	.069	—								
Waistline	-.004	.196	.062	.150	.846*	—							
PBL	.144	.038	.135	.141	-.308*	-.125	—						
PBF	-.122	-.030	-.124	-.129	.318*	.129	-.997*	—					
LMI	.050	.269*	.118	.165	.751*	.733*	.391*	-.380*	—				
SMI	.098	.334*	.213	.240*	.647*	.638*	.367*	-.357*	.884*	—			
T score	-.052	.082	.051	.090	.045	.178	.443*	-.491*	.353*	.304*	—		
Grip strength	.144	.182	.202	.253*	.195	.273*	.512*	-.515*	.543*	.568*	.409*	—	
Gait speed	.155	.173	.050	.075	.086	.018	.093	-.085	.146	.254*	.136	.421*	—

*BMI* body mass index, *PBL* percentage of body lean, *PBF* percentage of body fat, *LMI* lean mass index, *SMI* skeletal muscle mass index; *P <0 .05

**Fig. 4:**
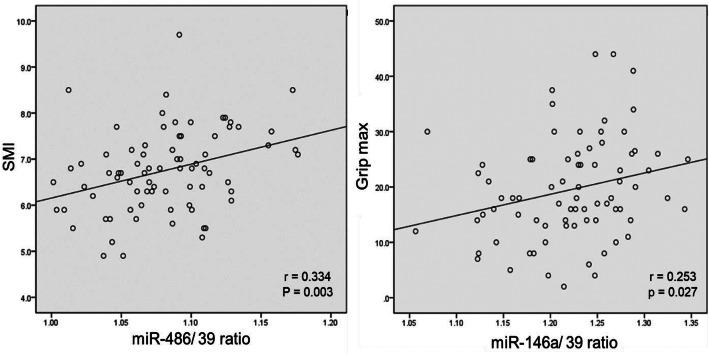
Association between: ashows the c-miR-486 and SMI (*r* = .334, *p* = .003); **b**shows the c-miR-146a and handgrip strength (*r* = .253, *p* = .027)

Both c-miR-486 and c-miR-146a were placed into a forward and stepwise, multivariate regression model that includes BMI, LMI, SMI, and handgrip strength. After adjustment, c-miR-486 level was meaningfully associated with SMI (*r* = 0.334, *P* = 0.003) whereas c-miR-146a level was significantly correlated to handgrip strength (*r* = 0.253, *P* = 0.027) (Fig. 4).

### The diagnostic accuracy of the sarcopenia

**Fig. 5:**
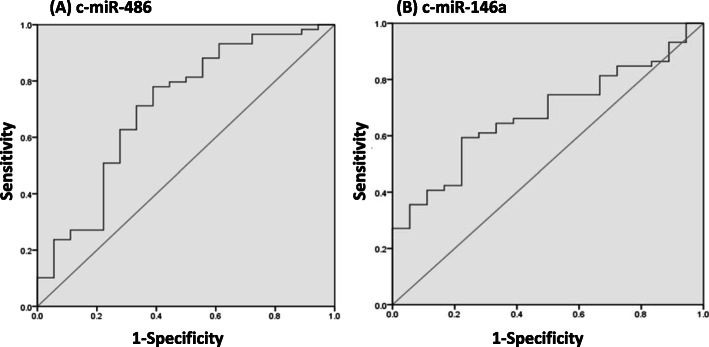
ROC curves analysis of c-miRNAs for predicting sarcopenia. **a** c-miR-486 AUC was .708 (95% CI: .561–.855, *p* = .008). **b** c-miR-146a AUC was .676 (95% CI: .551–.801, *p* = .024). ROC, receiver operating characteristic. AUC, areas under the curves. CI, confidence interval

The ROC curve analysis evaluated the diagnostic accuracy of c-miRNAs and sarcopenic parameters. The AUC in c-miR-486 was 0.708 (95% confidence interval, CI: 0.561 ~ 0.855, *P* = 0.008) with cut-off point was 0.391 (78% sensitivity, 61.1% specificity) (Fig. 5a). Moreover, the AUC in c-miR-146a was 0.676 (95% CI:0.551 ~ 0.801, *P* = 0.024) with cut-off point was 0.371 (59.3% sensitivity, 77.8% specificity) (Fig. 5b).

## Discussion

This study is the first to report that c-miR-486 and c-miR-146a serve as potential biomarkers of sarcopenia-related declines of muscle mass and strength, respectively. However, plasma lipid peroxide and inflammatory cytokines are not associated with sarcopenia-declined muscle mass and functions in the older adults.

Sarcopenia is likely by multifactorial contributors in sub-health conditions, includes muscular senescence, sedentary lifestyle, poor nutritional status, hormonal dysregulation, and pro-inflammatory status [8, 10]. Previous studies have focused on muscle size as the major cause of age-declined muscle dysfunction. However, loss of muscle mass plays a relatively minor role in age-declined muscle function [8, 10]. According to the ROC curve analysis for predicting sarcopenia in the present study, the c-miR-486 and c-miR-146a with sarcopenia are AUCs of 0.708 and 0.676 with significant differences, respectively. These findings imply that either c-miR-486 or c-miRNA-146a acts as diagnostic and potential biomarkers for the prediction of sarcopenia in the older adults.

The c-miR-486 is highly expressed in skeletal muscle, that directly targets Pax7 to promote myoblast differentiation [31]. It also reduces the expressions of PTEN and FoxO1a, in turn phosphorylating AKT and activating PI3K/AKT pathway [14, 24, 32]. Therefore, lowered c-miR-486 level observed from the sarcopenic older adults may represent as a result of progressive loss in muscle mass. The c-miR-146a serves as an anti-inflammatory miRNA, that negatively regulates the inflammatory response by targeting TNF receptor-associated factor 6 (TRAF6) and IL-1R-associated kinase (IRAK-1) to inactivate NF-κB in cytoplasm [6, 20, 23]. The miR146a also modulates cellular senescence, mitochondrial metabolism, and inflammation responses [12]. Conversely, downregulation of miR146a could accelerate the aging process and lead to immunosenescence [12]. In this study, sarcopenic older adults had lower c-miR-146a along with lesser handgrip strength, suggesting that poor muscle strength is associated with pro-inflammatory state in sarcopenic process.

Increasing evidence has demonstrated that aging process deteriorates mechanisms to maintain protein homeostasis and proteostasis. Proteostasis is the maintenance of protein homeostasis through mechanisms that involve the location, concentration, conformation, and turnover of individual proteins [3]. Decrease of c-miR-486 in the aging process could impair protein turnover of skeletal muscle, leading to the loss of contractile protein and accumulation of protein damage [14].

Age-related declines in mitochondrial function also contribute to the disturbance of proteostasis processes in skeletal muscle [19]. When mitochondrial dysfunction, the electron transport system leads to an imbalance, which results in the formation of reactive oxygen species (ROS). Furthermore, miR-146a regulates mitochondria of NOX4 protein expression, thus modulating cellular senescence and redox status [29]. Downregulated miR-146a is associated with exacerbated ROS production from mitochondria and oxidative damage in aging process [1]. In contrast, maintain mitochondrial function can facilitate mechanisms of proteostasis. Thus, mitochondrial dysfunction caused by downregulated miR-146a may depress energy production and impair skeletal muscle function under progression of sarcopenia [22].

### Limitation of the study

A limitation of this study is the lack of a control cohort of younger health subjects chosen base on the same criteria of exclusion. Because the ages of these three groups are similar, the main purpose of this study focuses on the effect of inflammation on sarcopenia in the elderly. Additionally, the cross-sectional study design is a major limitation of this study. The loss of muscle mass and poor muscle strength or physical performance in these older adult participants may be only partially attributable to physiological aging, and the influences of genetic selection, lifestyle, and nutritional status or the differences in other characteristics among the three groups cannot be excluded. Moreover, the current experimental results have not provided direct evidence to clarify how c-miRNAs regulate the sarcopenic processes in the older adults. Further longitudinal and interventional studies are needed in the future.

## Conclusions

This investigation clearly demonstrates that both myo (c-miR-486)- and inflammation (c-miR-146a)-related c-miR levels are positively associated with muscle mass, whereas only inflammation-related c-miR (c-miR-146a) level is directly related to muscle strength in the older adults. However, there are no significant relationships between sarcopenic parameters and plasma inflammatory cytokines. Hence, circulating myo-miRNA and inflammation-related miRNA are superior to inflammatory cytokines in plasma for serving as critical biomarkers of age-related sarcopenia.

## Data Availability

The datasets analyzed during the current study are available from the corresponding author on reasonable request.

## Abbreviations

AWGS: Asian Working Group of Sarcopenia
AUC: Area under the curve
BMI: Body mass index
c-miRs: circulating microRNAs
D: Dynapenic
EDTA: Ethylenediaminetetraacetic acid
IL-1β: Interleukin-1β
IL-6: Interleukin-6
IRAK-1: Interleukin-1 receptor-associated kinase
LMI: Lean mass index
MPO: Myeloperoxidase
N: Normal
PBF: Percentage of body fat
PBL: Percentage of body lean
ROC: Receiver operating characteristic
ROS: Reactive oxygen species
RT-qPCR: Real-time quantitative polymerase chain reaction
S: Sarcopenic
SMI: Skeletal muscle mass index
TNF: Tumor necrosis factor
TRAF6: Tumor necrosis factor receptor associated factor 6

## References

[1] Anderson EJ, Lustig ME, Boyle KE (2009). Mitochondrial H_2_O_2_ emission and cellular redox state link excess fat intake to insulin resistance in both rodents and humans. J Clin Invest.

[2] Baggish AL (2011). Dynamic regulation of circulating microRNA during acute exhaustive exercise and sustained aerobic exercise training. J Physiol..

[3] Balch WE, Morimoto RI, Dillin A, Kelly JW (2008). Adapting proteostasis for disease intervention. Science.

[4] Bohannon RW, Schaubert KL (2005). Test-retest reliability of grip-strength measures obtained over a 12-week interval from community-dwelling elders. J Hand Ther.

[5] Cortez MA, Bueso-Ramos C, Ferdin J, Lopez-Berestein G, Sood AK, Calin GA (2011). MicroRNAs in body fluids--the mix of hormones and biomarkers. Nat Rev Clin Oncol.

[6] Cornett AL, Lutz CS (2014). Regulation of COX-2 expression by miR-146a in lung cancer cells. RNA.

[7] Cruz-Jentoft AJ, Baeyens JP, Bauer JM (2010). Sarcopenia: European consensus on definition and diagnosis: report of the European working group on sarcopenia in older people. Age Ageing.

[8] Cruz-Jentoft AJ, Sayer AA (2019). Sarcopenia. Lancet.

[9] Czimmerer Z (2013). A versatile method to design stem-loop primer-based quantitative PCR assays for detecting small regulatory RNA molecules. PLoS One..

[10] Dhillon RJ, Hasni S (2017). Pathogenesis and Management of Sarcopenia. Clin Geriatr Med.

[11] Fan J, Kou X, Yang Y, Chen N (2016). MicroRNA-regulated proinflammatory cytokines in sarcopenia. Mediat Inflamm.

[12] Jiang M, Xiang Y, Wang D, Gao J, Liu D, Liu Y, Liu S, Zheng D (2012). Dysregulated expression of miR-146a contributes to age-related dysfunction of macrophages. Aging Cell.

[13] Kao TW, Chen WL, Han DS, Huang YH, Chen CL, Yang WS (2016). Examining how p16(INK4a) expression levels are linked to handgrip strength in the elderly. Sci Rep.

[14] Kirby TJ, McCarthy JJ (2013). MicroRNAs in skeletal muscle biology and exercise adaptation. Free Radic Biol Med.

[15] Limpawattana P, Kotruchin P, Pongchaiyakul C (2015). Sarcopenia in Asia. Osteoporosis and Sarcopenia..

[16] McGregor RA, Poppitt SD, Cameron-Smith D (2014). Role of microRNAs in the age-related changes in skeletal muscle and diet or exercise interventions to promote healthy aging in humans. Ageing Res Rev.

[17] Menghini R, Casagrande V, Federici M (2013). MicroRNAs in endothelial senescence and atherosclerosis. J Cardiovasc Transl Res.

[18] Metter EJ, Talbot LA, Schrager M, Conwit R (2002). Skeletal muscle strength as a predictor of all-cause mortality in healthy men. J Gerontol A Biol Sci Med Sci.

[19] Musci RV, Hamilton KL, Miller BF (2018). Targeting mitochondrial function and proteostasis to mitigate dynapenia. Eur J Appl Physiol.

[20] Park H, Huang X, Lu C, Cairo MS, Zhou X (2015). MicroRNA-146a and microRNA-146b regulate human dendritic cell apoptosis and cytokine production by targeting TRAF6 and IRAK1 proteins. J Biol Chem.

[21] Sasaki H, Kasagi F, Yamada M, Fujita S (2007). Grip strength predicts cause-specific mortality in middle-aged and elderly persons. Am J Med.

[22] Siegel MP, Kruse SE, Percival JM (2013). Mitochondrial-targeted peptide rapidly improves mitochondrial energetics and skeletal muscle performance in aged mice. Aging Cell.

[23] Singer JW, Fleischman A, Al-Fayoumi S, Mascarenhas JO, Yu Q, Agarwal A (2018). Inhibition of interleukin-1 receptor-associated kinase 1 (IRAK1) as a therapeutic strategy. Oncotarget.

[24] Small EM, O'Rourke JR, Moresi V, Sutherland LB, McAnally J, Gerard RD, Richardson JA, Olson EN (2010). Regulation of PI3-kinase/Akt signaling by muscle-enriched microRNA-486. Proc Natl Acad Sci U S A.

[25] Sourvinou IS, Markou A, Lianidou ES (2013). Quantification of circulating miRNAs in plasma: effect of preanalytical and analytical parameters on their isolation and stability. J Mol Diagn.

[26] Stenholm S, Tiainen K, Rantanen T, Sainio P, Heliövaara M, Impivaara O, Koskinen S (2012). Long-term determinants of muscle strength decline: prospective evidence from the 22-year mini-Finland follow-up survey. J Am Geriatr Soc.

[27] Tieland M, Trouwborst I, Clark BC (2018). Skeletal muscle performance and ageing. J Cachexia Sarcopenia Muscle.

[28] Valadi H, Ekström K, Bossios A, Sjöstrand M, Lee JJ, Lötvall JO (2007). Exosome-mediated transfer of mRNAs and microRNAs is a novel mechanism of genetic exchange between cells. Nat Cell Biol.

[29] Vasa-Nicotera M, Chen H, Tucci P, Yang AL, Saintigny G, Menghini R, Mahè C, Agostini M, Knight RA, Melino G, Federici M (2011). miR-146a is modulated in human endothelial cell with aging. Atherosclerosis.

[30] Wilson CM, Kostsuca SR, Boura JA (2013). Utilization of a 5-meter walk test in evaluating self-selected gait speed during preoperative screening of patients scheduled for cardiac surgery. Cardiopulm Phys Ther J.

[31] Xiao C, Rajewsky K (2009). MicroRNA control in the immune system: basic principles. Cell..

[32] Xu M, Chen X, Chen D, Yu B, Huang Z (2017). FoxO1: a novel insight into its molecular mechanisms in the regulation of skeletal muscle differentiation and fiber type specification. Oncotarget.

[33] Zhang T, Brinkley TE, Liu K, Feng X, Marsh AP, Kritchevsky S, Zhou X, Nicklas BJ (2017). Circulating MiRNAs as biomarkers of gait speed responses to aerobic exercise training in obese older adults. Aging.

